# 4D Flow MRI Velocity and Turbulence Mapping in Mild Valvular Heart Disease

**DOI:** 10.1002/jmri.70087

**Published:** 2025-08-20

**Authors:** Tamara Bianchessi, Chiara Trenti, Carl‐Johan Carlhäll, Tino Ebbers, Jan Engvall, Farkas Vanky, Federica Viola, Petter Dyverfeldt

**Affiliations:** ^1^ Division of Diagnostics and Specialist Medicine, Department of Health, Medicine and Caring Sciences Linköping University Linköping Sweden; ^2^ Center for Medical Image Science and Visualization (CMIV) Linköping University Linköping Sweden; ^3^ Department of Clinical Physiology Linköping University Hospital Linköping Sweden; ^4^ Department of Health, Medicine and Caring Sciences Linköping University Linköping Sweden; ^5^ Science for Life Laboratory Linköping University Linköping Sweden; ^6^ Department of Cardiothoracic Surgery Linköping University Hospital Linköping Sweden

**Keywords:** 4D flow MRI, turbulent kinetic energy, valvular heart disease

## Abstract

**Background:**

Valvular heart disease (VHD) commonly leads to the development of turbulent blood flow. Turbulent kinetic energy (TKE), measured with 4D flow MRI, may be a complement to current metrics for early identification of VHD.

**Purpose:**

To investigate TKE as a marker of VHD in relation to flow velocity and cardiovascular geometry.

**Study Type:**

Retrospective observational cross‐sectional.

**Population:**

Twenty controls and 106 subjects with VHDs, including mitral regurgitation, aortic regurgitation, pulmonary regurgitation, tricuspid regurgitation, and aortic stenosis.

**Field Strength/Sequences:**

Four‐dimensional flow MRI using a spoiled gradient‐echo phase‐contrast sequence with asymmetric 4‐point motion encoding at 1.5 or 3 T.

**Assessment:**

Time‐resolved segmentations of the left and right ventricles (LV, RV), atria (LA, RA), and aorta were performed. Total and maximum TKE, maximum and average velocity, and diameters were evaluated in each. Correlations between TKE, velocity, and diameter were assessed, along with group differences between VHD subjects and controls.

**Statistical Tests:**

Student's *t*‐test, Wilcoxon rank–sum test, chi‐squared test, Pearson's correlation, two‐way analysis of covariance. A *p* value < 0.05 was considered significant.

**Results:**

Total and maximum TKE correlated significantly with maximum velocity (*r* = 0.45–0.76) and averaged velocity (*r* = 0.22–0.44) and less strongly with diameters for aorta, LV, LA, and RV (*r* = 0.18–0.37). Compared to controls, total and maximum aortic TKE were significantly higher in aortic stenosis (3.8 vs. 1.6 mJ; 291.7 vs. 133.7 J/m^3^). Maximum LV TKE was significantly elevated in aortic regurgitation (106.6 vs. 91.8 J/m^3^). Total TKE was significantly elevated in LA for mitral regurgitation (1.1 vs. 0.6 mJ), in RA for tricuspid regurgitation (1.6 vs. 0.7 mJ), and in RV for pulmonary regurgitation (1.7 vs. 1.0 mJ).

**Data Conclusion:**

TKE is elevated in mild VHD. When evaluated alongside velocity as a marker for VHD, TKE may be more discriminative. Consequently, it has potential to be a hemodynamic marker of early VHD conveying complementary information to velocity.

**Evidence Level:**

4.

**Technical Efficacy:**

Stage 1.

## Plain Language Summary

This study explored how turbulent kinetic energy (TKE), a measure of blood flow disturbances, can help detect early signs of valvular heart disease. Using advanced cardiac MRI scans, TKE and blood flow velocity were measured in 126 subjects, of which 106 had mild valve problems and 20 had none. TKE values were higher in people with valve disease, even when the disease was mild, which was not seen with blood flow velocity measurements. These results suggest that TKE could be a useful additional tool for early identification of heart valve disease.

## 1 Introduction

Heart valves allow the efficient functioning of chamber circulation. Valvular heart disease (VHD) is prevalent in high‐income countries mainly as a result of degenerative valve disease (primary VHD) or as a result from pathologies in adjacent cardiac structures (secondary VHD) [1, 2]. Left‐sided VHD includes aortic valve stenosis (AS) with a global prevalence of 9.4 million patients, aortic valve regurgitation (AR), and mitral valve regurgitation (MR) with a prevalence of 24.2 million patients worldwide; AS is mainly caused by valve leaflet calcification, AR by aortic root dilation, and MR by valvular dysfunction or by being secondary to left ventricular dysfunction [1, 2, 3, 4]. On the right side of the heart, tricuspid valve regurgitation (TR) is often associated with pulmonary valve stenosis (PS) and regurgitation (PR), at times reflecting tetralogy of Fallot but more frequently being secondary to left‐sided heart disease [1, 5].

Current clinical imaging evaluation of VHD is primarily based on Doppler echocardiography, which grades valvular stenosis based on peak velocity measurements and valvular insufficiency based on the extent of the regurgitation jet and ventricular function [6]. Time‐resolved, three‐dimensional phase‐contrast MRI (4D flow MRI) is an alternative method that may provide complementary information through its three‐dimensional nature and access to a wide variety of hemodynamic parameters. Previous studies have demonstrated the capability of 4D flow MRI to quantify and visualize flow in both normal and diseased heart valves and have also demonstrated agreement between peak jet velocity and transthoracic echocardiography in aortic valve stenosis [7, 8, 9, 10].

In addition to velocity‐based parameters, 4D flow MRI permits estimation of turbulence intensity and turbulent kinetic energy (TKE) by exploiting the effects of velocity fluctuations on the amplitude of the phase‐contrast MR signal [11, 12]. Previous studies have shown that TKE measured with 4D flow MRI is a potential marker of several cardiovascular diseases in the aorta, pulmonary artery, or in one of the four cardiac chambers. For example, aortic TKE is elevated and associated with pressure loss in subjects with aortic stenosis [13, 14, 15, 16]. Further, mitral regurgitation causes higher left atrial (LA) TKE when compared to normal controls, and left ventricular (LV) TKE is elevated in subjects with different degrees of dilated cardiomyopathy when compared to controls [17, 18]. Finally, TKE may also be a useful marker for altered systolic flow jet and LV outflow tract anatomy in patients with hypertrophic cardiomyopathy [19]. In the right heart, TKE has been reported to be higher in the right atrium, right ventricle, and pulmonary artery in subjects with repaired tetralogy of Fallot when compared to normal controls [20, 21]. Based on the results of these previous studies, we hypothesize that TKE could also be a marker of mild‐to‐moderate VHD throughout the whole heart and may provide complementary information compared to velocity.

Thus, the aim of this study was to measure TKE and velocity with 4D flow MRI in mild VHD across the whole heart, to explore the relationship between TKE, velocity, and cardiac geometry, and to identify the extent to which TKE and velocity are altered in VHD.

## 2 Materials and Methods

This is a single‐center retrospective cross‐sectional study performed in line with the declaration of Helsinki. The study was approved by the regional ethical review board, and written informed consent was obtained from all subjects. The data analyzed in this study were collected for research purposes between 2011 and 2022.

### 2.1 Study Population

Our 4D flow MRI database was queried for whole‐heart datasets acquired with sequences that permit whole‐heart TKE estimation, and 126 subjects were identified from four different studies. The subjects were divided into a disease cohort (106 subjects) presenting with a variety of VHDs in combination with ischemic cardiomyopathy, idiopathic dilated cardiomyopathy, or left ventricular diastolic dysfunction and a control cohort (20 subjects) without any known cardiovascular disease (see Table 1). The control cohort consisted of volunteers without any known cardiovascular diseases that were recruited for previous studies. The cases with VHD were further classified into sub‐cohorts based on a conventional transthoracic echocardiography performed by ultrasound technicians prior to the MRI examination, using a Vivid E9 scanner (GE, Vingmed Ultrasound, Horten, Norway). 2D gray‐scale images were acquired for the parasternal and apical views, and Doppler images were acquired to investigate transvalvular flow in the aortic, mitral, pulmonary, and tricuspid positions. The echocardiography data were analyzed offline using the manufacturer analysis software (EchoPac, v. 112, GE Healthcare, Milwaukee, WI) by two investigators (C.‐J.C., J.E., more than 20 years of experience in reading echocardiography) according to standard recommendations [22]. Doppler velocities were used to identify and grade valvular stenosis (cut‐off value of 180 cm/s), and the extent of the diameter of the regurgitant jet on color Doppler was used to identify and grade valvular insufficiency. In this way, 233 valvular lesions were identified among the 106 cases.

**TABLE 1:** Cohort characteristics.

	Controls	VHDs sub‐cohorts				
		AS	AR	MR	PR	TR
*N*	20	24	77	75	14	43
VHD severity: mild/moderate	/	24/0	74/3	69/6	14/0	37/6
Females	9 (45%)	12 (50%)	25 (32%)	26 (35%)	11 (78%)	19 (44%)
Age [years]	35.5 [29.5, 39.25]	69.5 [65.5, 73.0]*	68.0 [64.0, 71.0]*	68.0 [63.5, 70.0]*	65.5 [60.8, 68.8]*	69.0 [65.5, 70.0]*
Weight [kg]	73.5 ± 6.4	79.3 ± 12.44	79.1 ± 13.4	78.2 ± 13.4	71.1 ± 11.5	78 [63.5, 87.5]
Height [cm]	168.0 ± 0.0	170.3 ± 8.6	172.2 ± 8.4	171.9 ± 8.3	168.8 ± 8.0	171.1 ± 9.7
BSA [m^2^]	1.8 ± 0.0	1.9 [1.7, 2.1]	1.9 [1.8, 2.1]	1.9 ± 0.2	1.8 [1.7, 2.0]	1.9 [1.7, 2.0]
Heart rate [bpm]	62.0 [59.25, 73.0]	62.5 [59.8, 72.2]	68.0 [62.0, 76.0]	69.0 [62.0, 82.0]	65.2 ± 12.3	66.0 [61.0, 71.5]
LV stroke volume [mL]	83.3 ± 25.3	82.6 ± 16.7	78.4 ± 15.3	77.7 ± 16.1	71.7 [65.5, 95.9]	78.8 ± 17.7
LVESVi [mL/m^2^]	41.1 [32.4, 45.8]	36.2 [31.3, 46.9]	36.2 [31.4, 43.1]	36.5 [31.3, 43.1]	32.4 [30.6, 42.8]	36.6 [31.1, 45.5]
LVEDVi [mL/m^2^]	82.3 [67.6, 101.3]	78.6 [69.2, 97.1]	76.8 [68.4, 84.9]	77.0 [68.6, 85.0]	72.7 [68.8, 95.1]	77.0 [68.8, 86.2]
RVESVi [mL/m^2^]	42.4 ± 13.9	38.0 ± 8.4	36.6 [33.1, 41.1]	36.7 [33.0, 41.7]	40.7 ± 10.9	36.7 [32.7, 44.1]
RVEDVi [mL/m^2^]	90.0 ± 30.2	76.0 ± 12.6	74.4 [67.8, 81.7]	74.9 [67.9, 82.3]	77.9 [72.8, 84.6]	76.6 ± 11.3

Abbreviations: AR, aortic regurgitation, AS, aortic stenosis; BSA, body surface area; LV, left ventricle; LVEDVi, left ventricle end diastolic volume indexed to BSA; LVESVi, left ventricle end systolic volume indexed to BSA; MR, mitral regurgitation; PR, pulmonary regurgitation; RVEDVi, right ventricle end diastolic volume indexed to BSA; RVESVi, right ventricle end systolic volume indexed to BSA; TR, tricuspid regurgitation; VHD, valvular heart disease.

* *p* value < 0.05.

### 2.2 MRI Data Acquisition and Post Processing

The datasets for the included subjects were obtained with a spoiled gradient‐echo phase‐contrast sequence with 2.8 mm isotropic spatial resolution and simple asymmetric 4‐point motion encoding with three orthogonal motion encodings and one fully flow‐compensated reference encoding on a Philips Achieva 1.5 T or Ingenia 3 T scanner between 2011 and 2022. Scan time was 10–15 min including navigator efficiency at 60 bpm. 4D flow MRI scan parameters aligned well with the current 4D flow consensus recommendations [23] (Table 2). All 4D flow MRI acquisitions were corrected for concomitant gradient field effects on the scanner, whereas phase wraps and background phase offsets were corrected in post‐processing [24, 25]. Time‐resolved 3D segmentations of the 4D flow data were generated with an in‐house deep learning tool for Datasets 3 and 4 and a multi‐atlas‐based method for Datasets 1 and 2 [26, 27]. Both tools used 4D flow MRI magnitude images as input. Atlas‐based segmentations of 80% of the data in Datasets 1 and 2 were used to train the deep learning tool in a previous study [26]. As an evaluation of the deep learning tool was not within the scope of this study, the fact that this approach biased the deep learning segmentations of the present study was ignored. Visual assessment and manual correction of the deep learning segmentations were performed by one observer (T.B., 2 years' experience in cardiovascular MR image processing). The atlas‐based segmentations were available from previous studies [26, 27]. The ascending aorta (AAo) was truncated at a standard location just proximal to the brachiocephalic trunk, which encompasses the region in which elevated TKE can be expected in subjects with less than moderate aortic stenosis, and the AAo diameter was estimated from the average cross‐sectional area taken over several planes located along an automatically generated centerline [15]. The equivalent diameter was used to estimate the size of non‐tubular structures. The equivalent diameter was estimated from the segmentation volume of the atria and ventricles by modeling each cavity as a sphere and deriving the diameter from the sphere's volume. The diameters were computed at the timeframe of peak average velocity and peak maximum velocity in each cardiac chamber. Left ventricular stroke volume was computed as the difference between the end diastolic and end systolic volumes obtained from the segmentations of the 4D flow data. BSA was computed with according to Du Bois formula [28].

**TABLE 2:** Scan parameters.

	Dataset 1 (cases)	Dataset 2 (cases)	Dataset 3 (controls)	Dataset 4 (controls)
Number of subjects	71	35	16	4
System	Philips 3 T MRI system (Ingenia CV, Philips Healthcare, Best, The Netherlands)	Philips 3 T MRI system (Ingenia CV, Philips Healthcare, Best, The Netherlands)	Philips 1.5 T MRI system (Achieva dStream, Philips Healthcare, Best, The Netherlands)	Philips 1.5 T MRI system (Achieva dStream, Philips Healthcare, Best, The Netherlands)
Sequence	SGRE (Spoiled Gradient Echo)	SGRE (Spoiled Gradient Echo)	SGRE (Spoiled Gradient Echo)	SGRE (Spoiled Gradient Echo)
Acquired and reconstructed spatial resolution	Isotropic ~2.8 mm^3^	Isotropic ~2.8 mm^3^	Isotropic ~2.8 mm^3^	Isotropic ~2.8 mm^3^
Slice orientation	Sagittal oblique covering the whole heart and thoracic aorta	Sagittal oblique covering the whole heart and thoracic aorta	Sagittal oblique covering the whole heart and thoracic aorta	Sagittal oblique covering the whole heart and thoracic aorta
Temporal resolution and number of reconstructed time frames	52.8 ms, 40 time frames	54 ms, 40 time frames	39.2 ms, 40 time frames	39.68 ms, 40 time frames
TR	4.4 ms	4.5 ms	4.9 ms	4.96 ms
TE	2.6	2.7	2.6	2.9
Flip angle	10	10	5	5
K‐space segmentation factor	3	3	2	2
Parallel acquisition technique	SENSE (3 in AP direction)	SENSE (3 in AP direction)	SENSE (2 in AP direction, 2 in RL direction)	SENSE (2 in AP direction, 2 in RL direction)
K‐space filling	Elliptical	Elliptical	Elliptical	Elliptical
VENC	120	120	150	120
Cardiac gating	Retrospective	Retrospective	Retrospective	Retrospective
Respiratory gating	Respiratory navigator gating using an inner window of 4 mm for 20% of the k‐space, and an outer window of 15 mm	Respiratory navigator gating using an inner window of 4 mm for 20% of the k‐space, and an outer window of 15 mm	Respiratory navigator gating using an inner window of 4 mm for 25% of the k‐space, and an outer window of 15 mm	Respiratory navigator gating using an inner window of 4 mm for 20% of the k‐space, and an outer window of 15 mm
Contrast agent	0.2 mmol/kg Gadovist, Bayer Schering Pharma AG	0.2 mmol/kg Gadovist, Bayer Schering Pharma AG	None	None

### 2.3 Hemodynamic Parameters

TKE was computed as:

(1) $$\mathrm{TKE}=\frac{1}{2}\cdot \rho \cdot \sum_{i=1}^{3}\sigma_{i}^{2}\;\left[J/m^{3}\right]$$

where ρ is the blood density, assumed to be $1060\;\mathrm{kg}/m^{3}$, $\sigma_{i}$ is the velocity fluctuation intensity expressed as intravoxel velocity standard deviation in three orthogonal directions. $\sigma_{i}$ in Equation (1) was computed as:

(2) $$\sigma_{i}=\frac{1}{k_{v}}\cdot \sqrt{2\cdot \ln\left(\frac{\left|S\right|}{\left|S_{i}\right|}\right)}$$

where $\left|S\right|$ and $\left|S_{i}\right|$ are the magnitude of the MR signal without and with motion encoding in direction *i*, respectively [11, 29]. $k_{v}=\frac{\pi }{\text{VENC}}$ represents the motion sensitivity.

The following velocity and TKE‐based parameters were computed in the aorta and cardiac chambers to gauge the hemodynamic effect of different VHDs. Total TKE (TKE_tot_ $\left[\mathrm{mJ}\right]$) was obtained by integrating TKE in each region for each time frame. Maximum TKE (TKE_max_ $\left[J/m^{3}\right]$) was defined as the maximum TKE in each region for each time frame. Average velocity (Vel_avg_) $\left[m/s\right]$ was computed as the average speed in the volume at each time frame, and maximum velocity (Vel_max_) $\left[m/s\right]$ as the maximum speed in the volume at each time frame. A median filter with a 3 × 3 × 3 kernel was employed to reduce noise in the estimation of TKE_max_. The TKE and velocity parameters were automatically quantified at the time‐point of peak early diastolic filling in the ventricles to reflect aortic and pulmonary regurgitation. Similarly, the same parameters were automatically obtained at peak ventricular systole for the atria and aorta to reflect aortic stenosis and mitral and tricuspid regurgitation. All velocity and TKE parameters were averaged around a ±2.5% window, that is, one time frame before and one time frame after the time‐point of interest to suppress noise effects. The time points were chosen to represent the moment in time when the deformed valvular geometry had the highest impact on blood flow [30, 31]. The Reynolds number was computed as:

(3) $$R_{e}\left[-\right]=\frac{\rho \cdot v\cdot D}{\mu }$$

where ρ is the blood density, assumed to be $1060\;\mathrm{kg}/m^{3}$, v is the blood speed in m/s, D is the diameter of the region of interest in meters, and μ is the dynamic viscosity of the fluid, assumed to be $4\cdot 1e^{-3}\;\mathrm{Pa}\cdot s$. *R*_*e*_ was evaluated for both Vel_avg_ and Vel_max_ with the corresponding, previously defined, diameters. All the hemodynamic parameters were computed using Python 3.9.18.

### 2.4 Statistical Analysis

Continuous variables are presented as mean ± SD when normally distributed, and as median [25th percentile, 75th percentile] when not. Categorical variables are presented as percentages. Differences between controls and cases regarding age, heart rate, and stroke volume were evaluated with a Student's *t*‐test or Wilcoxon rank–sum test, depending on the distribution of the variables. A two‐way analysis of covariance (ANCOVA) was computed to independently investigate differences in TKE and velocity measures between each of the VHD sub‐cohorts and the controls cohort; characteristics, such as age, that were significantly different in the two cohorts were included as covariates in the analysis to investigate their effect. A chi‐squared test was used to assess the difference in sex between the case cohort and the control cohort. Linear regression (Pearson test) was used to investigate the correlation between TKE_tot_ and TKE_max_ and Vel_avg_, Vel_max_, diameters, and Reynolds numbers for all subjects. A *p* value < 0.05 was considered significant. The statistical evaluation was carried out using Python 3.9.18 with the module statsmodels 0.14.1.

## 3 Results

### 3.1 Relationship Between Velocity, TKE and Diameter

Correlations for both TKE_tot_ and TKE_max_ with, respectively, Vel_avg_, Vel_max_, diameter, and Reynolds numbers are presented in Table 3. Vel_max_ showed higher correlations with both TKE measurements (*ρ* = 0.45–0.76) than Vel_avg_ (*ρ* = 0.22–0.44) in all regions, while diameters had lower correlation values (*ρ* = 0.18–0.37) with RA for both TKE measurements, not significant along with AAo and LV for TKE_max_. Reynolds numbers, combining diameters and velocity measurements, generally correlated with higher or equal *r* values compared to the corresponding velocity. Reynolds numbers computed with Vel_max_ presented higher correlations (*ρ* = 0.45–0.75) compared to one derived with Vel_avg_ (*r* = 0.24–0.65).

**TABLE 3:** Correlations between TKE measurements and other investigated variables in the study population. Values are presented as *r* and with a 95% CI.

TKE	Region	Vel_avg_ [m/s]	Vel_max_ [m/s]	Mean diam [m]	Max diam [m]	*R*_*e*_ (Vel_avg_, mean diam) [−]	*R*_*e*_ (Vel_max_, max diam) [−]
TKE_tot_ [mJ]	AAo	0.06 [−0.12, 0.23]	0.59 [0.46, 0.69]*	0.30 [0.13, 0.45]*	0.28 [0.11, 0.43]*	0.28 [0.11, 0.43]*	0.65 [0.54, 0.74]*
	LV	0.43 [0.28, 0.56]*	0.70 [0.60, 0.78]*	0.34 [0.18, 0.49]*	0.32 [0.15, 0.47]*	0.65 [0.53, 0.74]*	0.75 [0.66, 0.82]*
	LA	0.38 [0.22, 0.52]*	0.46 [0.31, 0.59]*	0.37 [0.21, 0.51]*	0.33 [0.16, 0.48]*	0.54 [0.40, 0.65]*	0.56 [0.43, 0.67]*
	RV	0.44 [0.29, 0.57]*	0.59 [0.46, 0.69]*	0.25 [0.08, 0.41]*	0.20 [0.03, 0.36]*	0.62 [0.50, 0.72]*	0.59 [0.46, 0.69]*
	RA	0.27 [0.09, 0.42]*	0.45 [0.30, 0.58]*	−0.01 [−0.18, 0.17]	0.06 [−0.12, 0.23]	0.24 [0.07, 0.40]*	0.45 [0.30, 0.58]*
TKE_max_ [J/m^3^]	AAo	0.22 [0.05, 0.38]*	0.76 [0.67, 0.83]*	0.04 [−0.14, 0.21]	0.04 [−0.14, 0.21]	0.27 [0.10, 0.42]*	0.59 [0.46, 0.69]*
	LV	0.42 [0.26, 0.55]*	0.63 [0.51, 0.73]*	0.15 [−0.02, 0.32]	0.09 [−0.08, 0.26]	0.52 [0.38, 0.64]*	0.56 [0.43, 0.67]*
	LA	0.32 [0.15, 0.47]*	0.56 [0.43, 0.67]*	0.22 [0.05, 0.38]*	0.22 [0.05, 0.38]*	0.45 [0.30, 0.58]*	0.64 [0.52, 0.73]*
	RV	0.26 [0.09, 0.42]*	0.63 [0.51, 0.73]*	0.18 [0.01, 0.34]*	0.20 [0.03, 0.36]*	0.32 [0.15, 0.47]*	0.64 [0.52, 0.73]*
	RA	0.36 [0.20, 0.50]*	0.63 [0.51, 0.73]*	−0.07 [−0.24, 0.11]	0.04 [−0.14, 0.21]	0.27 [0.10, 0.42]*	0.59 [0.46, 0.69]*

Abbreviations: AAo, ascending aorta; CI, confidence interval; LA, left atrium; LV, left ventricle; Max diam, diameter computed at peak Vel_max_; Mean diam, diameter computed at peak Vel_avg_; RA, right atrium; *R*_*e*_, Reynolds number; RV, right ventricle; TKE_max_, peak turbulent kinetic energy; TKE_tot_, total turbulent kinetic energy; Vel_avg_, peak average velocity; Vel_max_, peak maximum velocity.

* *p* value < 0.05.

### 3.2 Differences Between Cases and Controls

**FIGURE 1:**
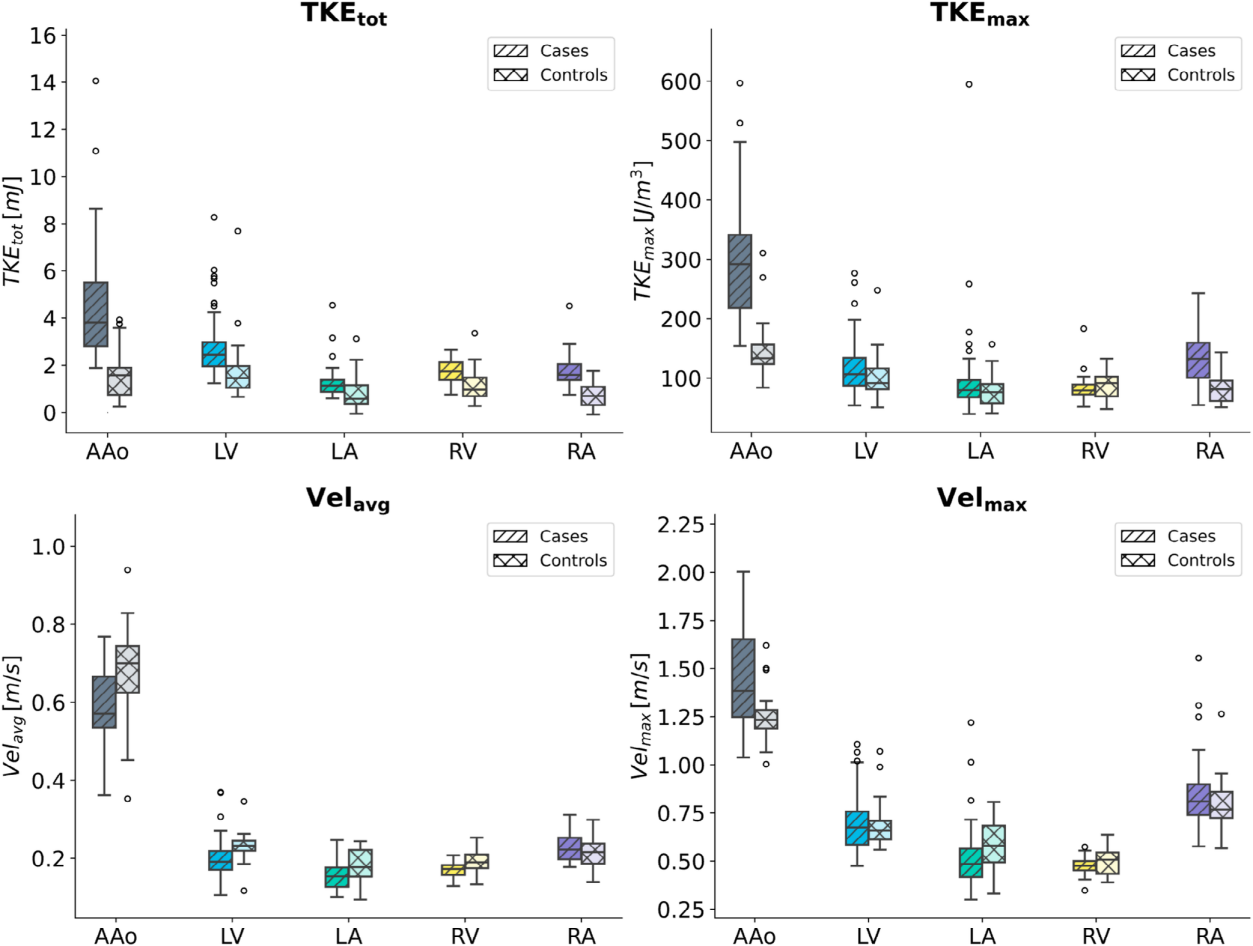
Box plots. Data distribution and differences between cases and controls for each region of interest and for each hemodynamic parameter, TKE_tot_, TKE_max_, Vel_avg_, Vel_max_. AAO, ascending aorta; LA, left atrium; LV, left ventricle; RA, right atrium; RV, right ventricle.

**FIGURE 2:**
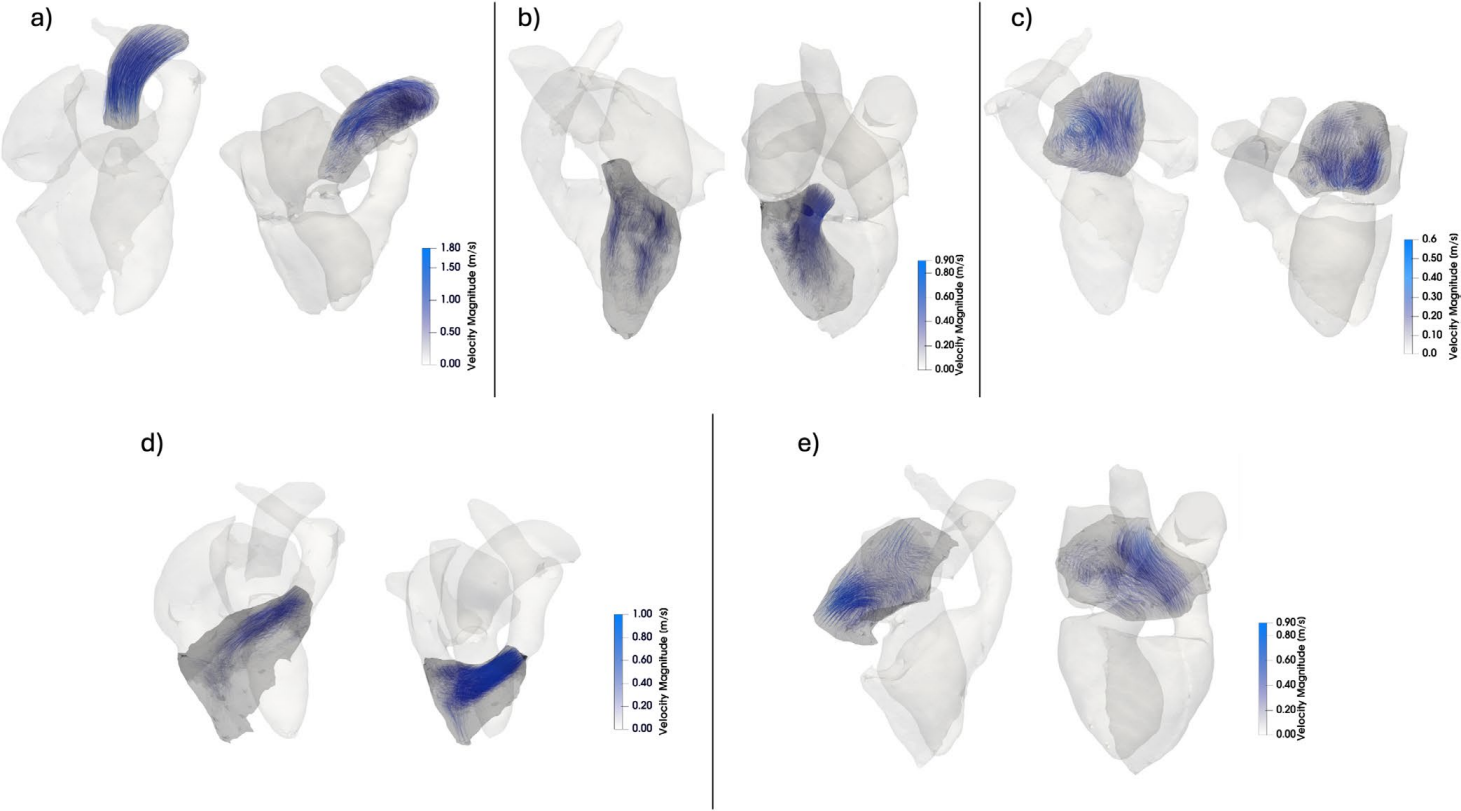
Streamline visualization of flow patterns (blue color scale). (a) Ascending aorta at systole for a normal control (left) and a subject with aortic stenosis (right), (b) left ventricle at diastole for a normal control (left) and a subject with aortic regurgitation (right), (c) left atrium at systole for a normal control (left) and a subject with mitral regurgitation (right), (d) right ventricle at diastole for a normal control (left) and a subject with pulmonary regurgitation (right), (e) right atrium at systole for a normal control (left) and a subject with tricuspid regurgitation (right). The color scale is the same for each pair of images.

**FIGURE 3:**
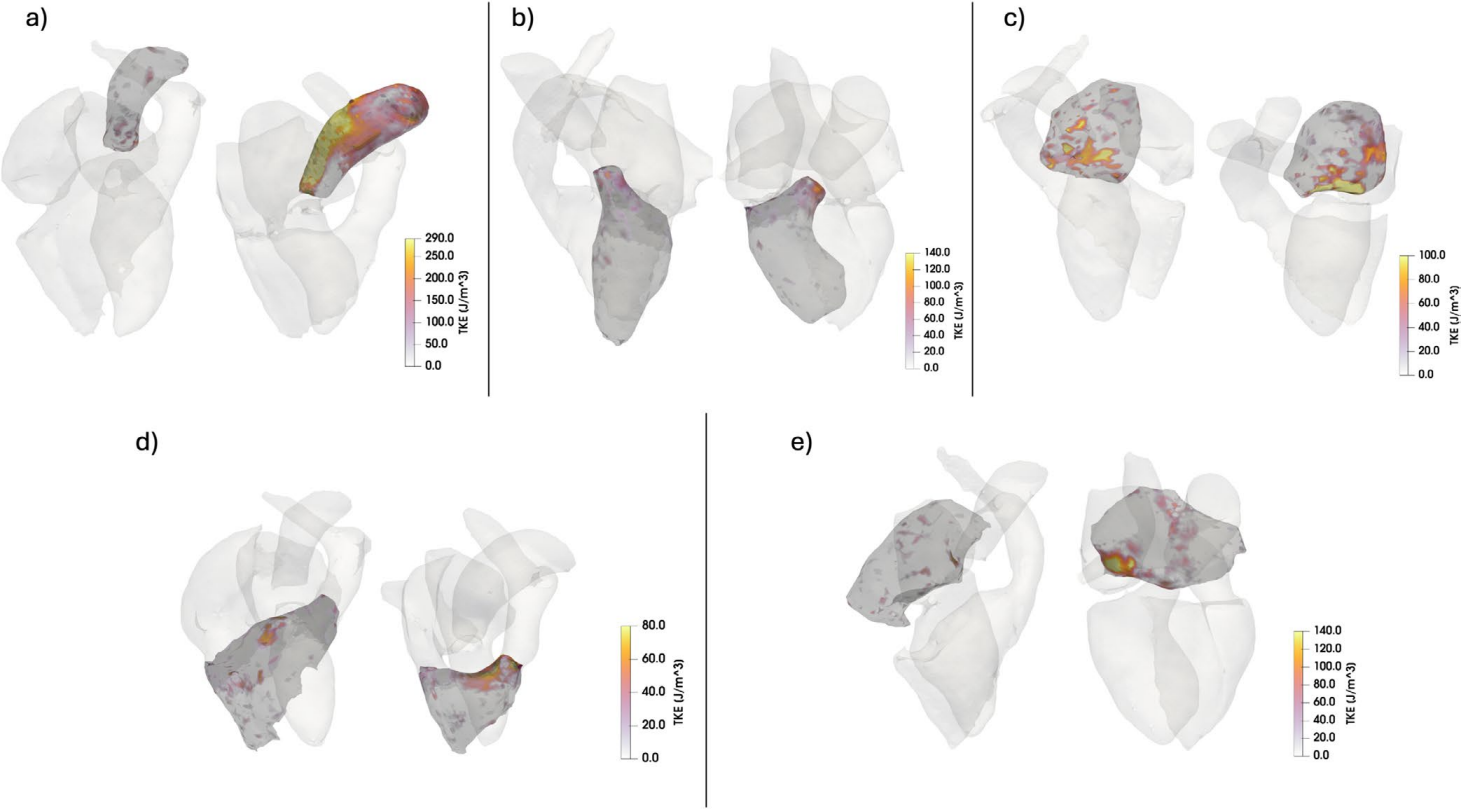
Volume rendering of turbulent kinetic energy (TKE) (red to yellow color scale). (a) Ascending aorta at systole for a normal control (left) and a subject with aortic stenosis (right), (b) left ventricle at diastole for a normal control (left) and a subject with aortic regurgitation (right), (c) left atrium at systole for a normal control (left) and a subject with mitral regurgitation (right), (d) right ventricle at diastole for a normal control (left) and a subject with pulmonary regurgitation (right), (e) right atrium at systole for a normal control (left) and a subject with tricuspid regurgitation (right). Note that TKE typically manifests as more clearly different than velocity, underlining the results in Table 4. The color scale is the same for each pair of images.

The hemodynamic parameters for the four cardiac chambers and the AAo are presented in Table 4 along with the results from the ANCOVA analysis. Corresponding boxplots for the hemodynamic parameters are shown in Figure 1. TKE_tot_ (3.8 vs. 1.6 mJ) and TKE_max_ (291.7 vs. 133.7 J/m^3^) in the AAo at systole were significantly higher in cases with aortic stenosis than controls. No significant differences in velocity were found in cases with aortic stenosis and controls after correcting for the effect of age. In the LV at diastole, both TKE_max_ (106.6, vs. 91.8 J/m^3^) and Vel_avg_ (0.2 vs. 0.2 m/s) were significantly higher in cases with aortic regurgitation than in controls, but age had a significant effect in both cases as shown by the significant interaction term in the ANCOVA analysis. In the LA at systole, TKE_tot_ was significantly higher in cases with mitral regurgitation than in controls (1.1 vs. 0.6 mJ). In addition, Vel_max_ (0.5 vs. 0.6 m/s) was significantly lower in the controls, but this difference was affected by age (interaction term). RV TKE_tot_ at diastole was the only hemodynamic parameter that differed significantly between cases with pulmonary regurgitation and controls (1.7 vs. 1.0 mJ). Similarly, RA TKE_tot_ at systole was the only hemodynamic parameter that differed significantly between cases with tricuspid regurgitation and controls (1.6 vs. 0.7 mJ). Example visualizations of velocity and TKE in a control without VHD and in cases with different forms of VHD are shown in Figures 2 and 3, respectively; the subjects are the same for each corresponding region in the two figures.

**TABLE 4:** ANCOVA analysis in hemodynamic measures between cases and controls.

Region	VHD	Hemodynamic measures			ANCOVA analysis—coefficient, CI [2.5%, 97.5%]		
			Controls	Cases	VHD	Age	Interaction (VHD:age)
AAo	AS (*n* = 24)	TKE_tot_ [mJ]	1.6 [0.7, 1.9]	3.8 [2.8, 5.5]	2.789 [0.232, 5.346]*	0.011 [−0.60, 0.082]	−0.0624 [−0.210, 0.086]
		TKE_max_ [J/m^3^]	133.7 [123.7, 156.4]	291.7 [218.0, 341.2]	143.744 [39.557, 247.932]*	0.500 [−2.377, 3.377]	0.086 [−6.003, 6.175]
		Vel_avg_ [m/s]	0.7 ± 0.1	0.6 ± 0.1	0.032 [−0.082, 0.146]	−0.004 [−0.0007, −0.001]*	0.006 [−0.010, −0.012]
		Vel_max_ [m/s]	1.3 ± 0.1	1.5 ± 0.3	−0.336 [−1.213, 0.538]	−0.002 [−0.010, 0.007]	0.009 [−0.006, 0.024]
LV	AR (*n* = 77)	TKE_tot_ [mJ]	1.5 [1.0, 2.0]	2.4 [2.0, 3.0]	−2.605 [−5.994, 0.784]	−0.022 [−0.067, 0.022]	0.0610 [−0.001, 0.123]
		TKE_max_ [J/m^3^]	91.8 [81.2, 116.4]	106.6 [87.5, 134.4]	−102.157 [−211.352, −7.038]*	−0.704 [−2.140, 0.731]	2.031 [0.047, 4.014]*
		Vel_avg_ [m/s]	0.2 [0.2, 0.2]	0.2 [0.2, 0.2]	−0.177 [−0.293, −0.061]*	−0.002 [−0.003, −0.001]*	0.003 [0.001, 0.005]*
		Vel_max_ [m/s]	0.7 [0.6, 0.7]	0.7 [0.6, 0.8]	−0.060 [−0.178, 0.058]	0.002 [−0.001, 0.005]	0.010 [−0.004, 0.015]
LA	MR (*n* = 75)	TKE_tot_ [mJ]	0.6 [0.4, 1.2]	1.1 [0.9, 1.4]	−1.605 [−2.307, −0.351]*	0.004 [−0.021, 0.029]	0.009 [−0.027, 0.044]
		TKE_max_ [J/m^3^]	76.4 [57.9, 90.1]	79.8 [67.8, 96.7]	−147.132 [−344.115, 39.851]	−0.1204 [−2.513, 2.272]	2.424 [−0.967, 5.814]
		Vel_avg_ [m/s]	0.2 [0.2, 0.2]	0.2 [0.2, 0.2]	−0.026 [−0.147, 0.095]	−0.001 [−0.002, 0.001]	0.001 [−0.002, 0.002]
		Vel_max_ [m/s]	0.6 [0.6, 0.6]	0.5 [0.5, 0.5]	−0.156 [−0.595, 0.282]*	−0.005 [−0.010, 0.001]*	0.003 [−0.005, 0.011]*
RV	PR (*n* = 14)	TKE_tot_ [mJ]	1.0 [0.7, 1.5]	1.7 [1.4, 2.1]	0.891 [0.138, 1.643]*	−0.012 [−0.033, 0.010]	−0.009 [−0.063, 0.044]
		TKE_max_ [J/m^3^]	91.7 [69.4, 102.7]	79.2 [72.6, 89.3]	−100.896 [−223.088, 21.297]	0.007 [−0.924, 0.937]	1.541 [−0.481, 3.563]
		Vel_avg_ [m/s]	0.2 ± 0.0	0.2 ± 0.0	−0.020 [−0.146, 0.106]	−0.001 [−0.002, 0.000]	0.001 [−0.002, 0.002]
		Vel_max_ [m/s]	0.5 ± 0.1	0.5 ± 0.1	0.008 [−0.322, 0.621]	−0.001 [−0.003, 0.002]	−0.001 [−0.006, 0.005]
RA	TR (*n* = 43)	TKE_tot_ [mJ]	0.7 [0.4, 1.2]	1.6 [1.4, 2.0]	1.310 [0.605, 2.014]*	−0.009 [−0.030, 0.011]	0.001 [−0.041, 0.041]
		TKE_max_ [J/m^3^]	83.9 ± 23.8	135.7 ± 48.2	−51.615 [−207.406, 104.175]	−0.592 [−2.362, 1.178]	1.799 [−0.922, 4.520]
		Vel_avg_ [m/s]	0.2 [0.2, 0.2]	0.2 [0.2, 0.3]	0.066 [−0.068, 0.200]	0.001 [−0.000, 0.003]	−0.001 [−0.004, 0.001]
		Vel_max_ [m/s]	0.8 [0.7, 0.9]	0.8 [0.7, 0.9]	−0.330 [−0.984, 0.323]	−0.003 [−0.011, 0.004]	0.007 [−0.004, 0.018]

*Note*: Hemodynamic measures as derived from the 4D flow MRI measurements are presented along with the ANCOVA analysis with the corresponding coefficient, confidence interval, and *p* value for each of the variables used for the statistical analysis. VHD indicates a significant difference between cases and controls when only considering the presence of the VHDs after adjusting for age; age indicates a significant difference between cases and controls when only considering the effect of age and adjusting for the presence of VHD; interaction (VHD:age) indicates a significant difference between cases and controls when considering together the presence of VHD and age.

Abbreviations: AAo, ascending aorta; AR, aortic regurgitation; AS, aortic stenosis; CI, confidence interval; LA, left atrium; LV, left ventricle; MR, mitral regurgitation; PR, pulmonary regurgitation; RA, right atrium; RV, right ventricle; TKE_max_, peak turbulent kinetic energy; TKE_tot_, total turbulent kinetic energy; TR, tricuspid regurgitation; Vel_avg_, peak average velocity; Vel_max_, peak maximum velocity; VHD, valvular heart disease.

* *p* value < 0.05.

## 4 Discussion

Normal functioning valves are, in essence, check valves that help the heart and the main vessels maintain blood flow at conditions considered laminar under normal conditions, but the definition of flow features considered normal is challenging due to the complex nature of normal cardiovascular flow [32]. Distortions to normal cardiovascular geometry, such as those in VHDs, can cause elevated turbulence intensity; therefore, turbulence is a potential early marker of several cardiovascular diseases [33].

Supporting the notion that the presence of turbulent blood flow is a marker of cardiovascular disease, the main finding of the present study was that 4D flow was more discriminative of small changes in TKE compared to velocity‐based measures in asymptomatic VHD. When compared to MRI‐based velocity measurements, TKE measurements are robust to changes in spatial resolution, thus permitting reliable measurement at lower resolution, and consequently in less time, than MRI velocity [34]. With the increasing use of 4D flow MRI for various clinical purposes and increasing availability of deep learning‐based automatic segmentations, TKE quantification could augment MRI exams with complementary information on early signs of VHD.

The findings on TKE in mild VHD across the whole heart highlight interesting differences compared to velocity. Cases with mild aortic stenosis with elevated ascending aortic flow velocity at Doppler echocardiography did not have elevated velocity at 4D flow MRI but did have elevated TKE. This suggests that mild aortic stenosis with subtly elevated velocity in the AAo, not resolved by 4D flow velocity mapping, triggers velocity fluctuations detectable by 4D flow TKE mapping. TKE values for both controls and cases with aortic stenosis were slightly lower compared to those previously observed in similar cohorts, whereas velocity values were similar to those in previous studies [13, 15, 35].

Importantly, while peak velocity is a key metric for grading stenosis, it is not useful for assessing regurgitant lesions. This was reflected in our findings, where peak velocity was not consistent in all regions in discriminating between regurgitant VHD and controls. In contrast, TKE did discriminate between regurgitant VHD and controls, suggesting that TKE may serve as a more sensitive marker for the presence of regurgitant lesions. This supports our hypothesis that TKE could be a marker of mild‐to‐moderate VHD throughout the whole heart and highlights its potential role in grading the hemodynamic severity of regurgitant VHD beyond conventional metrics such as regurgitant volume. Results on LV TKE at diastole in cases with mild aortic regurgitation are scarce. The current study showed higher TKE_max_ in the LV in cases with aortic regurgitation compared to controls, but this difference was affected by both the presence of aortic regurgitation and its interaction with age. No significant differences were observed in TKE_tot_, despite the possible influence of dilated cardiomyopathy in the cohort, meaning that the regurgitant fraction of the blood through the aortic valve prompted elevated TKE value locally but not overall in the whole chamber. None of the patients had obstructed hypertrophic cardiomyopathy, which is known to impact TKE [19]. There was also a significant difference in Vel_avg_, which was affected by the disease, age, and the combined effect of the two. In the LA, the values for young controls were in line with those in previous studies [21, 36]. In the present study, a significantly higher TKE_tot_ was observed in the LA at systole in cases with mitral regurgitation, confirming that mild‐to‐moderate mitral regurgitation is associated with elevated TKE in the LA [17]. Vel_max_ was also lower in cases with mitral regurgitation, and it was significantly affected by age. Similarly, there was higher TKE_tot_ in the RV at diastole in cases with pulmonary regurgitation than controls, a result that is in line with a previous study [21]. In the RA, the only significant difference between cases and controls was observed for TKE_tot_, which was higher in cases with tricuspid regurgitation, also in line with a previous study in patients with tetralogy of Fallot [21].

To improve the understanding of TKE in normal cardiovascular blood flow and in mild VHD, the relationships between TKE and velocity, diameter, and Reynolds number were also explored in cases with mild VHD and controls. There was a moderate to strong positive correlation between TKE and velocity in chambers of the heart and aorta. TKE_max_ was more strongly correlated with Vel_max_ than Vel_avg_, suggesting that local peak velocity can more confidently be used to infer regions with suspected elevated TKE. Mean diameter computed at peak values for Vel_avg_ and Vel_max_ was overall weakly positively correlated with Vel_avg_ and was only significant for LA and RV at Vel_max_. TKE_tot_ correlation with LV diameter was in line with a previous study reporting peak *E*, which usually represents the highest velocity during diastole [18]. These findings suggest that diameter plays a role in the development of turbulence in the cardiovascular system, although not as much as velocity. For the Reynolds number, the correlation with TKE increased when both Vel and diameter correlated with TKE but decreased when one of the two did not, demonstrating that TKE, velocity, and diameter provide independent, largely complementary, information.

### 4.1 Limitations

First, the retrospective observational cross‐sectional nature of this study limited the availability of data. Larger, age‐matched cohorts and the use of identical imaging protocols in all subjects may increase the possibility of identifying further differences between the control group and each of the VHDs. An alternative to only using controls that are free from VHD is to include cardiac chambers not immediately affected by the valve disease as “control chambers.” As the impact of forward or backward propagating effects of VHD, as well as compensatory remodeling effects in other chambers, on parameters such as TKE have not been investigated, we decided not to include such “control chambers” in the present study. Future studies are warranted to explore the extent to which these effects impact hemodynamic parameters such as TKE. Second, controls were significantly younger than cases with VHD. This was accounted for by adjusting the age variable with the ANCOVA analysis and allowing exploration of the impact of age on both TKE and velocity. Third, the VHD cohort included mainly cases with mild or mild‐to‐moderate VHD severity with a relatively small number of subjects in some VHD categories; some cases also presented a concomitant cardiomyopathy, but TKE correlated only weakly with diameter measurements, thus suggesting that the effects observed were mainly caused by VHD. At last, the controls cohort and the VHD cohort were acquired at different field strengths (1.5 and 3 T, respectively) both with and without contrast, thus impacting SNR. Nevertheless, while a reduced SNR increases uncertainty in TKE estimation, bias in TKE measurements due to the rectified noise floor occurs when TKE is excessively high relative to the SNR and is likely not an issue in this study where TKE was low relative to the VENC [12].

## 5 Conclusion

This retrospective observational cross‐sectional study suggests that TKE may be a useful hemodynamic marker of mild VHD, providing complementary information to that derived from 4D flow MRI‐based velocity measurements. When compared to TKE, velocity was more strongly affected by age; thus, reducing the possibility of detecting mild VHD based on velocity alone, as is commonly performed with echocardiography.
